# Healthy Aging: Comparative Analysis of Local Perception and Diet in Two Health Districts of Côte d’Ivoire and Japan

**DOI:** 10.3389/fragi.2022.817371

**Published:** 2022-04-25

**Authors:** B. Bonfoh, B. V. Koné, Y. D. Koffi, T. Miyama, Y. Fujimoto, G. Fokou, J. Zinsstag, R. Sugimura, K. Makita

**Affiliations:** ^1^ Centre Suisse de Recherches Scientifiques en Côte d’Ivoire, Abidjan, Côte d’Ivoire; ^2^ Université Félix Houphouët Boigny, Abidjan, Côte d’Ivoire; ^3^ Rakuno Gakuen University, Hokkaido, Japan; ^4^ Swiss Tropical and Public Health Institute, Basel, Switzerland; ^5^ University of Basel, Basel, Switzerland

**Keywords:** healthy aging, perception, diet, Japan, Côte d'Ivoire

## Abstract

**Context:** Good health and longevity depend on dynamic interactions between biological, social, psychological, and environmental factors. Aging is globally a big challenge, particularly with the demographic transition, including population growth, and an emerging burden to society. Knowledge, behavior, diet, and consumption of animal source food were related to aging and emerged as the key factors modulating healthy aging.

**Objective:** The study was designed to understand the main healthy aging factors, such as knowledge, social network, and diet of elders, and to derive mutual learning from it for healthy aging.

**Methods:** A qualitative approach has been applied to explore health-related knowledge, attitude, and diet of elders from Ebetsu (Japan) and Tiassalé (Côte d’Ivoire) health districts, using focus group discussions and comparative context analysis between high- and low-income countries.

**Results:** The study shows that living longer is a common feature of people in Japan compared to Côte d’Ivoire, where the life expectancy is still low. Both groups of elders have social networks that support them, and both offer their gained experience to society. While Japanese elders depend on pension and insurance for income and medical treatments, Ivorians depend mostly on their children and social network in old age. The worries of elders differ between the two regions. In Ebetsu, elder members of the community are concerned about the future burden they pose for the younger generation if they develop ill-health, making them more resilient to aging. In Taabo, elders are considered to be culturally and socially useful to the society. Elders in Ebetsu pointed out that for healthy aging, education on diet at a younger age, physical activities, and access to basic social services are the key aspects. This was not observed in Taabo’s context. Being inactive and dependent on others were described as the most worrying situations for elders in Ebetsu, as it is perceived to increase the risk of non-communicable diseases and anxiety. Elders in Ebetsu have good knowledge on what constitutes a healthy diet, and they believe that diversifying their diet, reducing portions, and substituting red meat with good animal and vegetable proteins are best eating practices to maintain good health. In Côte d’Ivoire, the diet is imbalanced and the whole family consumes the same meal made mainly with high-energy staples and little protein. However, it is observed in both societies that adopting a good diet is very expensive.

**Conclusion:** The consciousness of aging is universal, but healthy aging varies according to the social systems, education, and knowledge on diet transition. Physical activities, protein–energy balance in diet, and social networks are the key for healthy aging in both contexts. The challenge is to find ways to increase knowledge regarding healthy aging and to strengthen the support system so that healthy aging becomes affordable.

## Introduction

People worldwide are living longer with an increased life expectancy because of the advancement of technology and public health interventions (Cutler and Miller 2005). This shift in the demographic structure of a country’s population toward older ages, known as “population aging,” started in high-income countries (HIC). In this global demographic transition, problems related to aging are affecting both HIC and low- and middle-income countries (LMIC).

In Japan, the proportion of population older than 65 years increased from 7.1% in 1970 to 26.3% in 2015 (MIC, 2021). In LMICs like Côte d’Ivoire in West Africa, this trend is also increasingly observed, with LMICs experiencing the greatest age structural change recorded in history (Mondal and Shitan 2014).

By 2050 many countries will have a similar proportion of elders as Japan, and more elders will be living in LMICs where health and social services are limited (Holzmann et al., 2009). The health status of the African population has improved significantly, resulting in an increase in life expectancy overall (WHO, 2018). However, this longevity coexists with the development of chronic and non-communicable diseases (NCDs, some linked to consumption patterns and lifestyle (Breuil and Euller-Ziegler, 2004; Hoogenveen et al., 2010), creating a costly burden for younger generations and health systems. Hence, healthy ageing and longevity remain an objective to be achieved in both in HIC and LMIC. Therefore, people longevity and health depend on successful dynamic interactions between biological, psychological, social, and environmental factors. Diet and physical activities appear to be the most accessible and modifiable factors to modulate ageing and, above all, to allow the prevention of certain emerging diseases like type II diabetes, obesity and arterial hypertension (Gordon et al., 1977; Ferry, 2010; Sgarbieri and Pacheco, 2017). Good knowledge and positive attitudes towards an active lifestyle and healthy eating in terms of quantity and quality are essential for good ageing (Martin, 2001; Marsman et al., 2018). Diet and nutritional behaviour regulate the physiological processes of ageing and determine healthy ageing (Ashima, 2004; Roberts and Rosenberg, 2006). Furthermore, animal source food play an important role in nutrition, metabolism and pathology, and its consumption should be controlled during life (Neumann et al., 2002). Food diversification to some extent and the modulation of the frequency of consumption of certain foods allow avoiding overloads in the pre-aging phase and reduce the risk of NCDs and their impact on aging. Several studies have addressed the nutrition of children and women, but only few studies have considered the diet of the elderly, particularly, those suffering from NCDs in the African context.

In Côte d'Ivoire, the life expectancy is about 56.8 overall (Milleliri et al., 2021) and 55 years in rural areas (HDSS data, 2015), and people around these ages are increasingly suffering from NCDs. They face protein deficiency in their diet, and the local food system is not diversified enough and dominated by high-energy diets (Dindé et al., 2017). Cultural factors, food restrictions and taboos, and lack of nutritional education have a considerable impact on people’s eating habits. These conditions increase the risk of the emergence of several NCDs and compromise healthy aging. Learning from HICs’ experience can help improve aging conditions for elders in LMICs. This study is part of a qualitative analyses based on direct observation and focus group discussion aiming to provide information on the elders’ knowledge, nutrition practices, health conditions, and social and environmental risk factors. While our study intended to provide information that could help design an intervention for healthy aging in Africa, the qualitative design allowed for mutual learning among research partners involved.

## Materials and Methods

### Study Area and Period

This comparative study took place in two areas at different time periods. The first study was conducted in Ebetsu, Hokkaido prefecture, Japan, from October to December 2018. Ebetsu is a city of 187.57 km^2^ located next to Sapporo, on the island of Hokkaido. The total population of Ebetsu was 119,086 people (1 May 2017) living in 56,325 households with a density of 630 people per km^2^. The Hokkaido prefecture is characterized by agriculture and had a food self-sufficiency rate of 214 and 196% at production value and calorie bases, respectively, in 2018 (MAFF, 2021). Ebetsu is divided into two zones, (i) the urban continuum of Sapporo, the economic zone, and (ii) the agriculture and industrial zone, producing rice, vegetables, and livestock, and where most of the population is educated in the two zones.

The second study took place from October to December 2020 in Taabo, a small town with a mix of urban and rural areas, in the south-centre of Côte d’Ivoire, approximately 150 km from the economic Abidjan and 60 km south of the political capital Yamoussoukro. Taabo is, in the health and demographic surveillance system (HDSS), composed of a small town, 13 villages, and over 100 hamlets. At the end of 2020, the total population was 49,000 inhabitants living in 6,707 households. The Taabo prefecture is characterized by crop production, which is the main socio-professional activity, and secondarily livestock activities. Most of the inhabitants are illiterate (72%). The population is young, as seen in most LMICs, and 45.5% are children under 15 years old, while elders only constitute 4.6% (Koné et al., 2015).

### Study Design

The study used a qualitative approach to collect and analyze data in Ebetsu and in the HDSS of Taabo. In both countries, people from the oldest section of the population, adjusted for the local life expectancy, were selected. In Ebetsu (Japan), we approached the local elderly (above 60 years) groups who are organized in different associations and who meet to discuss their problems and have periodic social events. The presidents of those associations were approached to get the willingness of their members to participate. In Taabo (Côte d’Ivoire), we have used the database of the demographic surveillance site to draw the list of elders (between 55 and 80 years). The enumerators assisted in contacting them in their location when the villages were selected. In Ebetsu, six focus group discussions (FGDs) were conducted with a total of 54 elders, including 24 men and 30 women. Each group was composed of nine participants: four men and five women. In Taabo, we conducted 10 FGDs with elders ranging from 55 to 80 years of age. However, eight with completed information were used for analysis. Each group was composed of three men and four women while considering gender and multilingual specificities.

For both sites, after a short introduction of participants and presentation of the study objectives, the discussions centered around four main themes (i) personal perception of aging; (ii) knowledge on healthy aging; (iii) risk factors for aging and available services for the elderly people; and (iv) elders’ diet with a focus on animal source food.

The questions on healthy aging focused on their lifestyle, their daily activities, the existing care systems, the special care received, and the main diseases to which they are exposed. Further discussion items focused on the organization and services for elders with an emphasis on their role in the society, the family organization around them, the type of support, the challenges, and the socio-economic burden linked to aging. Regarding elders’ diet and its influencing factors, we focused on the types of food, consumption patterns, supplements provided, and food accessibility.

### Data Collection Method

Qualitative data were collected during group discussions with the respondents, using a semi-structured interview guide (Supplementary Material: Guide for focus group discussion). In Ebetsu, group discussions were organized and conducted in Japanese by trained PhD students in epidemiology and nutrition. Each discussion lasted a maximum of 1 hour. Respondents were male and female participants between 60 and 90 years. The respondents from each focus group were able to provide quantitative data estimating the portion in weight (gram) and volume (liter) of the different dishes they consume (Supplementary Material: Quantitative information). In Taabo, group discussions lasted 1 hour and were mostly conducted by PhD students in biomedical nutrition and social science in French and sometimes in the local language (Baoulé or Malinké). Saturation was used to determine the sample size for focus group discussion as with the life expectancy we did not have too many elders in the range set by the WHO. Thus, the principle of information saturation, which results in the redundancy of responses as the focus groups progress, was the type of saturation used. Of the eight focus groups conducted, the first five were characterized by a variety of information gathered from participants on the main themes of the study. Subsequently, the last three focus groups were characterized by a redundancy of information already obtained during the five previous ones. Following that, we had reached the principle of saturation of information on the main themes of the study, which we discussed with the elderly persons in Taabo. A total of 71 respondents (39 women and 32 men) were finally recruited in Taabo. Some participants from each group were able to talk about their health condition but were not able to estimate the portion in weight (gram) and volume (liter) of the consumed dishes, like in Ebetsu. During all group discussions, major themes mentioned by participants were written on a post-it in English in Japan and in French in Côte d’Ivoire, and the discussions were recorded using a digital voice recorder. Deep discussion on specific items made it possible to reach a consensus among participants on the formation of food groups and ranking of food preferences. In both sites, to reduce potential bias in participants’ responses, we used triangulation technique by asking negative expressions to ensure consensus or collecting opposite opinions.

### Data Management and Analysis

The recordings were transcribed into Microsoft Word (Microsoft Corporation, Redmond, WA, United States), and the relevant verbatim was listed in Japanese and translated from Japanese into English by students who had a good command of both languages. In the context of Taabo, the recordings followed the same process of transcription from Baoulé or Malinké to French. For quality control, recorded sections were randomly chosen and the content compared to the transcribed text to verify that the translation was as close as possible to the original answers provided by the participants. The verbatim of the interviews and important conversations was coded in MAXQDA 16 (VERBI GmbH, Berlin, Germany) using a list of codes elaborated from key themes of the study (education, worries, elder’s organization, activities, food consumption habit, type of food and diseases). This qualitative analysis software enabled the management and coding of large sets of qualitative data and facilitates linking it to direct observations. Data from initial codes were then grouped into main categories identified for further content analysis. These categories were coded and reflected the perception of elders about aging; social life and organization of elders; knowledge and influencing factors of a healthy diet; perception of a healthy diet; and typology of sources of proteins consumed. The French was translated into English after coding and analysis of the data.

### Ethical Approval

The study received ethical approval from the Rakuno Gakuen University Ethical Committee in Japan (approval number: 18-4, 2018 October 29) and the National Committee for Ethics in Life Science and Health of Côte d’Ivoire (N/ref: 097-19/MSHP/CNESVS-kp). All participants provided written informed consent before being enrolled in the study. Participants could leave the study at any time without further obligation.

## Results

The perception of aging varied according to social, economic, and cultural factors. Life expectancy in Japan was higher compared to Côte d’Ivoire, a fact perceived by elders from Ebetsu to be due to good medical infrastructures, social support, and insurance. Based on the focus group discussions, the following three major themes related to healthy aging emerged: social life and network, autonomy and health risk worry, and finally, nutritional education and physical activities.

### Elders’ Perception, Social Life, Network, and Support

In Ebetsu, most elders stated that they belonged to associations or non-governmental organizations (NGO), enjoying social activities such as seminars, physical activities, games, educating young people, gardening, providing assistance to more vulnerable elders, joint lunches and dinners, hobbies, and volunteer work. Elders who are members of associations mentioned that they play an important role in knowledge and experience exchange, physical activities, and mutual support. They reported that the social network helped them to overcome many challenges such as difficulties in walking, cooking, and snow removal and also dependence and living with dementia for some of them. They noted that they receive support from several sources such as the city council, residence associations, their families, and care insurances and can easily access supportive services and visiting nurses. As indicated by a participant in a group discussion in Ebetsu, “over 65 year old people go directly to the health facility and receive necessary care” (woman in Ebetsu). However, they recognized that the support is not enough to satisfy their needs, especially for lonely elders. As they asserted during discussions, “Insurance is very expensive compared to the pension. The insurance automatically takes their part from the pension, which leaves only a little amount for people to spend. More support is needed for elders living alone” (woman in Ebetsu). They stated that organizational support to elders is critically important in winter when they are the most vulnerable, but elders were generally reluctant to bother other people and use the food assistance. We observed that in Taboo, such infrastructure and organizations for elders and service provision are lacking.

Despite their age, data from both sites showed that elders see themselves as particularly useful to the society, far from considering themselves only as a burden for the younger generations. Their perception is that they still constitute an important social category in their community. Their contribution is mainly important in supporting their own families in childcare in both Ebetsu and Taabo and specifically for cultural events in Taabo. In Ebetsu, participants stated that external housekeeping help is hardly affordable. For that reason, “when children are working, the elderly takes care of the grandchildren” (woman in Ebetsu). They perceive their expertise is also required concerning social problems and political activism where they play an important role because of their “experience and knowledge”. In Taabo, we observed that elderly people live with their families in the same household and share the same resources. They report to eat with family members and do not have a specifically adapted diet. They mentioned social interactions and group meetings to help manage some community issues or to organize social activities such as weddings, baptisms, funerals, and local justice. Contrary to their Ebetsu counterparts, elders in Taboo reported that they leave a largely sedentary life without an infrastructure providing opportunities for physical activities except walking. Most of them stated that they mainly depend on their children for food, money, and care as they do not have either pension or insurance. They recognized during discussions that, “as you get older you do not work as much as you used to, you stay at home all day long and even if you go to work it is just a bit … When you get to that stage of life where you do not have the strength anymore, it is the children who must take care of you. You are at home, and they take care of you (man in Taabo).

### Elders’ Autonomy, Health Risks, and Worries

Elders in both sites highlighted the need for healthy aging to maintain their personal autonomy. Participants from both sites agreed that to maintain a healthy condition, elders require social and medical services, which will help them to maintain an active life and promote a healthy life. Thus, from a medical perspective, healthy aging requires access to quality care with the aim to keep elders healthy and, if possible, support them in nursing as caring costs are expensive.

Results showed that elders in Ebetsu were worried about their children and younger generation and thought it necessary to teach them how to live healthily. They were also aware that they might increasingly constitute a burden for society in terms of low productivity. They perceived that the proportion of elders is becoming larger. They feared that “living longer in bed is not good and almost all people in that situation are not happy” (woman in Ebetsu). They justified their unhappiness with their belief that younger people do not have a good perception of aging because they think they need to pay too much for elders through the social security system. Elders in Ebetsu were fully aware of the risks of diseases related to their lifestyle and are cognizant of risk management strategies. They are worried that their lifestyle could cause diseases such as cancer, hypertension, diabetes, knee ache, backache, and osteoporosis with a risk of bone fractures. To avoid those diseases, they emphasized monthly health check-ups of blood pressure and body weight in conditions of easy access to health services.

On the other side, in Taabo, the elders are concerned that nothing is being done to accompany and plan aging. They recognize that all things they did when they were younger have an impact on their current health. They would like the younger generation to pay more attention on their diet to minimize the borne diseases at elderly stages. As there is no insurance or pension for most elderly people, they see themselves as a burden on their families. It is therefore difficult for them to demand a special diet linked to their state of health to prevent the onset of illness. They are content to be a moral and cultural support for their family.

### Healthy Aging Factors (Education/Physical Activities)

In Ebetsu, elders stated that the enabling factors of healthy and good aging are peaceful environment, good air, formal education on healthy life, good nutrition, and regular physical exercise. “Healthy food was taught to us when we were young” (woman in Ebetsu). They expressed the necessity to teach young generations about diet and their role in the family. One of them regretted, “talking about food and health with elder is very important thing, I should have had well-balanced diet from a younger age” (woman in Ebetsu). In the Taabo locality, the diet and the habits that result from the elderly are strongly dependent on the existing food and the ability to obtain it, as they lacked formal education at a younger age. Thus, without knowledge, they do not always consider the health dimension of what they eat but rather what is available and accessible to satisfy their appetite and recover strength.

### Food Typology of Elders’ Food

The diet structure and preferences of elders in Ebetsu are ranked in diet groups using the coding system. Table 1 provides the perceived benefit and risk of each type of food. Elder recommended increasing vegetable intake and reducing or avoiding sugar, cake, sausage, ham, wiener, and instant food. They report reducing animal protein carbohydrates and avoiding spicy and salty food because of high blood pressure. In addition, in Taabo, the elders recommended that the meat consumed by the elderly people should be well-cooked to be easily chewable, and their diet should be more oriented toward light and easily digestible foods.

**TABLE 1:** Food preferences, ascribed benefits, and negative association in Ebetsu.

Food preference rank	Food Group	Ascribed Benefit	Negative association
1	Fermented food products (*miso soup*, *natto*, yoghurt)	Aids digestion, well balanced, soft to preserve teeth	Has little taste
2	Fish, vegetables, seaweed, mushroom	Vegetable proteins, aids digestion	-
3	Milk and dairy products (cheese)	Aids digestion, soft to preserve teeth, provides minerals and vitamins	-
4	Chicken, egg, pork	Replaces red meat	-
5	Rice and other carbohydrates (moderate)	Energy, minerals	Risk of hyperglycemia
6	Green tea, barley tea, *aojiru*	Aids digestion	-
7	Beef, lamb	Prevents weakening bones and muscles	Risk of hypertension and hyperglycemia
8	Supplements: vinegar, garlic, proteins pills, iron, Vit C, blueberries, *shijimi*, collagen, calcium	Good for health	-

miso soup = vegetable soup with fish soup stock and soybean paste; natto = fermented soybean with Bacillus natto; aojiru = fresh green vegetable squeezed juice; shijimi = clam, clam extract.

**FIGURE 1:**
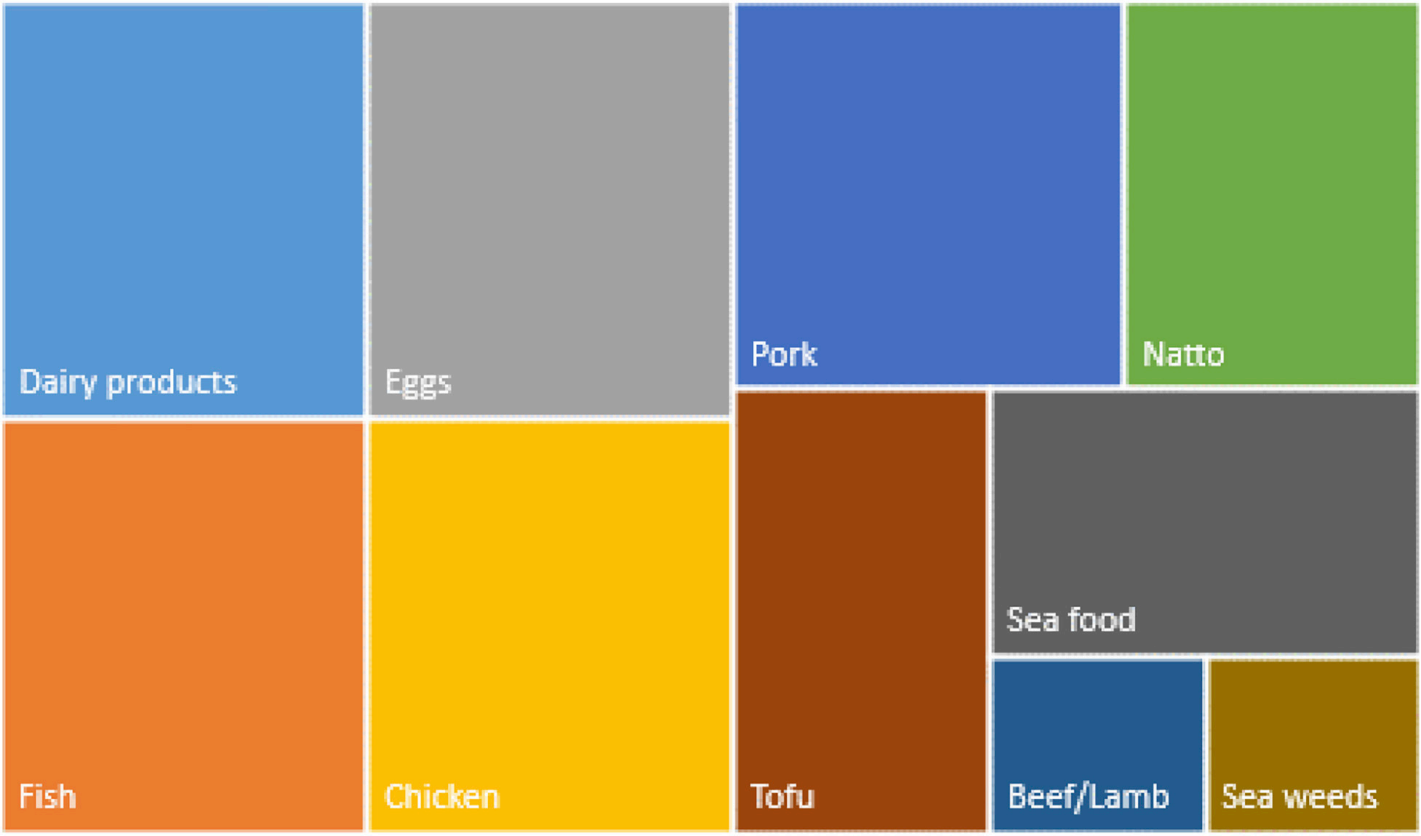
Preference ranking and most cited proteins sources consumed by elders (vertical size: most cited in the once or six times in the focus groups, horizontal size = high preference rank from 1 = low to 5 = high).

It appears that elders preferred fermented food products (*miso soup*, *natto*, and yoghurt), followed by fish, vegetables, and seaweed, while milk and dairy products emerged third. Chicken, eggs, and pork came fourth. We observed that the consumption of some products such as beef and lamb decrease with age while others like fish are consumed more. Compared to when they were young, the participants attested to have increased their fish intake with a ratio of meat to fish of 1:5. They reported to prefer vegetables cultivated in their own gardens, including tomato (*Solanum lycopersicum*) containing lycopene which is considered good for health. One elder advised applying the Japanese acronym *Ma-Go-Wa-Ya-Sa-Shi-I* as an awareness formula, which literally means “grandchildren are kind”). In detail, “*Ma*” represents “*mame*”, which is beans, “*Go*” for sesame, “*Wa*” for seaweed, “*Ya*” for vegetable, “*Sa*” for fish, “*Shi*” for mushroom, and “*I*” for tubers and roots. This provides a short acronym abbreviating the foods an elder should eat to stay healthy. They also feel it is important to balance their social and family connectivity and the need to be selective in their feeding choices. “Most of the time we eat what the entire family eats, but we avoid salty food and pay attention to sugar because of diabetes” (old woman in Taabo). In Ebetsu, they observed that even though it is expensive to get good quality foods, they avoid instant and processed food because of additives and high calories. They reported that red meat is consumed monthly with a lot of vegetables known as “*Jingiskan*,” which is a popular cuisine in Hokkaido prefecture, using a hot dome-shaped plate to cook lamb or mutton meat and later simmer vegetables in the soup extracted from vegetables, herbs, and meat in the peripheral pool part. The most frequently consumed products as mentioned by informants are poultry products, fish, and milk products, followed by soy products and seaweeds. Red meat appears as the fourth group of food commonly consumed by elders. The rank of products is given in Figure 1.

**FIGURE 2:**
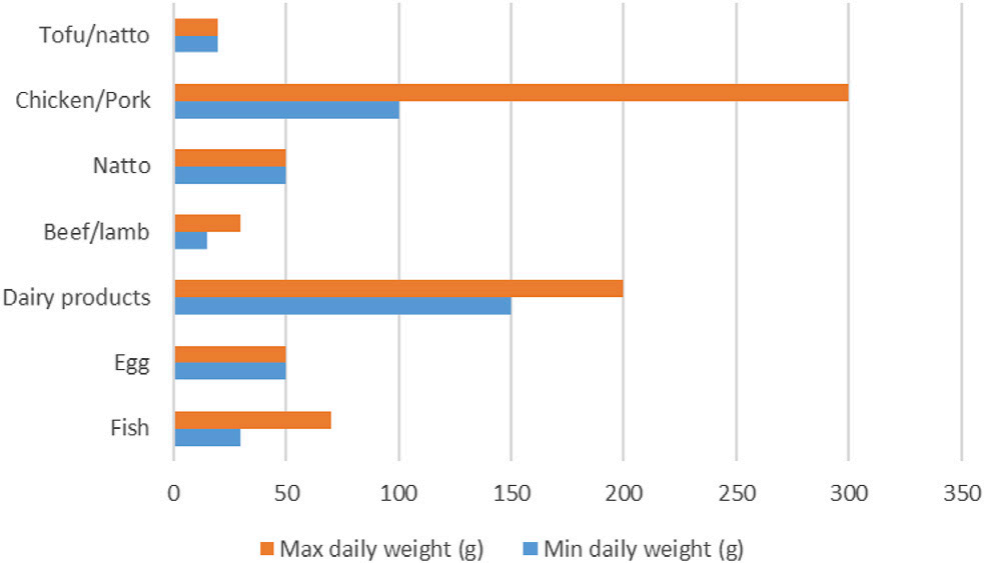
Range of daily average quantity of protein foods consumed by elders in Ebetsu.

A description of consumption habits of elders in Ebetsu shows that they are increasingly replacing animal proteins such as red meat (beef and lamb) with vegetable proteins in different dishes. Most products they consumed were fermented (fermented *natto*, cheese, and yoghurt). The most frequent proteins were eggs, chicken, tofu, *natto*, and dairy products, followed by fish, pork, beef, and lamb (Figure 2). Their preference leans toward fresh products that are consumed in small portions (size of palm). They supplement their food regularly with vinegar and vitamins and consume less salt, sugar, alcohol, fatty foods, and red meat (beef and lamb).

According to the respondents, the main reason for paying attention to their diet was to reduce the risks of developing all NCDs. Of proteins products, the quantity consumed in grams is inversely proportional to the frequency of consumption. Tofu and *natto* are consumed daily in small quantities (20 g), while red meat (lamb and beef) is consumed weekly or monthly (<400 g) but also with vegetables.

In Taabo, the diet expression depicts the food system and reflects the main food consumed. Elders say they eat to be alive and tend to cite high-energy food (staple) followed by protein (supplement). Their food type is mainly constructed around energy and not protein indicating the low consumption of proteins. In Ebetsu elders eat to be healthy and site proteins (staple) followed by energy (supplement). It appears from Table 2, that elders in Taabo do not have much choice but consume available and affordable food. Availability and capacity to access foods are more important determinant factors than the preference of foods. The main proteins ranked by elders are bush meat and fish. This is followed by the food group of poultry/chicken/egg, while red meat (beef, sheep, and goat) emerged third and as the most expensive group (consumed during festivals and ceremonies only). Compared to the Ebetsu society, in Taabo, food consumption patterns differ between social groups. These differences correspond generally to their agricultural practices, and food habits are specific to each group. The Baoulé group is more oriented toward the consumption of tubers such as yams, cassava, and plantain, which corresponds to their main agricultural practices, while the Malinke and people from neighboring countries are more oriented toward the consumption cereals such as maize, rice, and millet.

**TABLE 2:** Rank of the main diet groups in Taabo.

Rank	Food Preference and Choice Group	Perceived Role and Benefit to Elders	Perceived Risk to Elders Health
1	Cereals, tubers, and roots cooked and consumed with mixed sauce, vegetables, and spices	Energy to stay active, fill the stomach, and reduce hunger	-
1a	Foutou banana/yam/cassava	Provides energy and strength	-
1b	Toh (maize, millet)	Light and easy to digest	-
1c	Rice	Local rice from local crops is perceived as good for health	Industrial rice is of low quality for elders’ health
1d	Attieké (fermented cassava couscous)	Energy	Source of constipation
2	Proteins	Provides pleasure and prolongs life when smoked	Fresh meat many not be good
2a	Bush meat and meat (from an animal that has died without being slaughtered, or pork, bush animal)	Most accessible meat	Prohibited by law or because of religion, allergy, and social origin of elders
2b	Fish (fresh, dry), snails	Activates the appetite and gives strength and energy when consumed in times of illness	-
2c	Poultry/chicken/egg	Helps to soften the skin and reduce wrinkles on the body for elderly people	-
3	Red meat (beef, sheep, goat)	Promotes the increase of blood in the body and provides strength	-
4	Shellfish	Ate according to ethnic group and social origin, strengthens bones	-
5	Spices	Provide taste and enhance digestion	
5a	Cube-maggi	Enhances the taste of the sauce in absence of meat (high cost)	Source of diseases (hypertension)
5b	Soumara (fermented beans from *Parkia biglobosa* tree)	Control and balance hypertension disease	-
6	Petit cola (*Garcinia kola* is a fresh bitter cola nut)	Control sexual weakness and aphrodisiac. Helps fight against small diseases (body aches, stomach-ache, dizziness, nausea, and hypertension)	-

**FIGURE 3:**
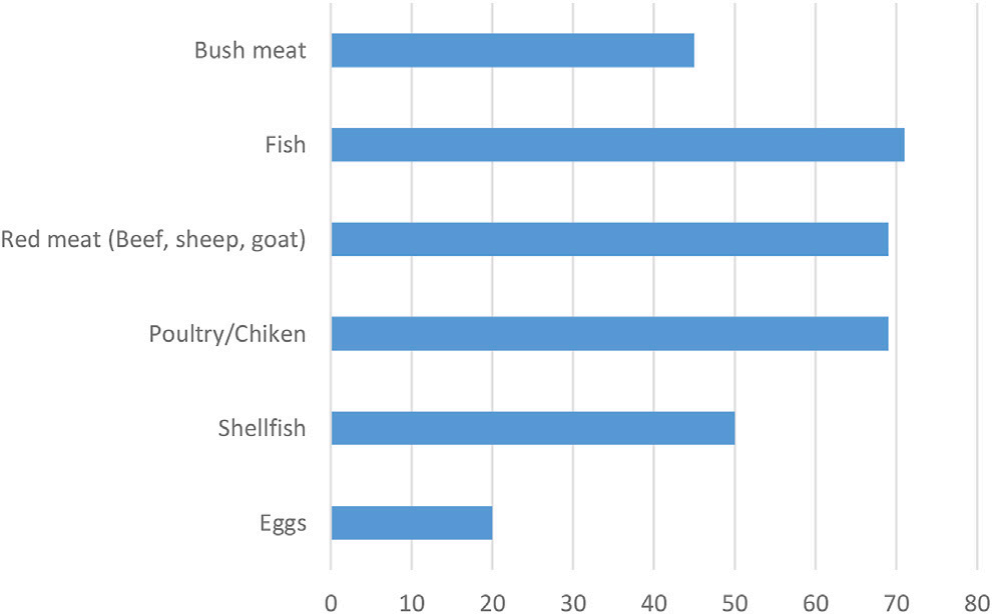
Percentage of animal protein in the diet consumed in Taabo (as number of times cited) by elders in Taabo.

As indicated in Figure 3, in Taabo, the description of consumption habits of elders showed that they were still consuming mostly fish, poultry/ chicken products, and bush meat compared to shellfish and red meat (beef, sheep, and goat).

### Aging and Healthy Diet and Lifestyle

For a healthy diet, elders in Ebetsu consider that both quality and quantity matter. Eating too much is not good for health, especially for those needing good teeth to chew food. Controlling the quantity of food ingested has become one of their daily habits. As they said, “We don’t want to leave unfinished dishes, so we don’t order large portions” (70-year-old Japanese woman). They reported, “avoiding eating until completely full and generally stopping eating when 80% full” (woman in Ebetsu). They also report paying attention to what they eat. They described the quality of preferred food as follows: “We eat mainly home-made food and use chopsticks as it is good exercise for the brain and prevents to eat more” (woman in Ebetsu). She also thinks that “hot food is not good for the optic nerve”.

They perceived imported products as being of bad quality and risky for their health even though they are cheaper. But the accessibility to high-quality food is perceived as expensive and not accessible to elders who rely on a tiny pension for their subsistence. They also recognized that at a certain age, they should have different eating habits to increase their life expectancy. Thus, they pay attention to salt, fatty food, and sugar, and avoid dishes based on meat, too spicy food, alcohol, and tobacco. “We used to drink or smoke, but now we stopped. Healthy food habits were taught by elders when we were young” (80-year-old man in Ebetsu). Consumption of healthy food such as fermented soybeans and well-balanced dishes combined with physical exercise such as walking 3,000–10,000 steps per day appeared as important risk management strategies by the elderly people. This is accompanied by preferential services for that specific category of people. For example, there are some advantages to fitness clubs: “We use fitness clubs and for people around 60 years old, there is a discount to motivate them” (62-year-old woman in Ebetsu). In Taabo, the factors influencing the diet are restriction of food for medical (NCDs) reasons (salt, oil, chilli, bouillon, etc.), personal factors (allergy, indigestion, etc.), and cultural reasons (forbidden, prohibited, or taboos). Most restrictions are linked to the religion (such as animals that died without being slaughtered and pork), sometimes to allergy (catfish) and cultural norms and ethnic origin (snails and bush meat). As an informant asserted, “we don’t eat the same things and there are things we don’t eat either because of our religion, our culture, or because we are not familiar with the type of food in question” (56-year-old woman in Taabo). Healthy diet is perceived by elders in terms of eating enough food to satisfy hunger. It is also a diet that respects the prescriptions of medical staff for sick people, or food that is in line with their traditions and norms. Elders consider it good to eat a diversified and balanced diet instead of eating modern or imported dishes (pasta, salads, potatoes, and rice). For one elder from Taabo, “when I eat, I just want to be full, it is not about quality. We often do not have the capacity to pay for the little good things to eat, even though they may be good for our health”) (61-year-old man in Taabo).

## Discussion

This study is part of a qualitative analysis based on direct observation and focus group discussion aiming to provide information on the elders’ knowledge, nutrition practices, health conditions, and social and environmental risk factors.

### Dynamics and Determinants of Aging in Japan and Côte d’Ivoire

The average life expectancy is 84 years in Ebetsu, while the average life expectancy in Taabo is only around 57 years. The increase of poverty in Côte d’Ivoire since the 1980s and 2000s and the failure of health policies have led to a considerable reduction in life expectancy in the country (Goba, 2017). The difference in life expectancy in Japan is certainly explained by lifestyle, access to services, and the country’s economic situation. Only 4.6% are over 60 years old in Taabo (Côte d’Ivoire) and only 2.45% of the population in 2014 was 65 years old (Coulibaly and Tuo, 2017) in Côte d’Ivoire.

This low life expectancy in Côte d'Ivoire is explained by the persistence of nutritional deficiencies and infectious diseases on one side and the emergence of other chronic metabolic disorders constitute a double burden detrimental to developing countries (Wells et al., 2020). This coexistence is associated with a low level of economic development, urbanization, poor quality food, and poor living conditions (Lobstein and Cooper 2020). In addition to various deficiencies, overnutrition may lead to chronic diseases related to nutrition in general, and both hypertension and diabetes mellitus are major public health problems in the developing countries. Seventy-nine percent of deaths worldwide attributed to chronic diseases occur in developing countries (Budreviciute et al., 2020). Nutritional problems are common in poor people whose diets are primarily based on starchy foods and plants (Adesogan et al., 2020).

In addition to NCDs, we note protein malnutrition in people over 70 years of age, weight loss and body mass index and albumin levels below the recommended threshold, indicating malnutrition (Doukoure et al., 2020). The presence of malnutrition, whatever the determinant(s) or nutrient(s) responsible, leads to elders with fragile health. Inadequate eating habits and behavior have nutritional consequences on the health of the elderly. It emerges from this study that the food choices of the elders were affected by their ability to cope with the cost of high-quality food, which is generally the most expensive in both countries. The drop in income of these people due to retirement makes access to quality food difficult because of the cost. The economic resources affect the nutritional choice of the elders (Dean et al., 2011), and in the Ivorian case, the socio-economic context particularly limits food diversification that is expressed in what they eat.

In the context of Taabo, there is no difference between the young and the elderly in terms of food consumption. Most of the elders live in their families and share the same food without mentioning specific needs. Although they felt the need for a lighter and more diversified diet adapted to their age, the economic constraints of the household force them to adopt the same food regimen as the rest of the family. In the context of Ebetsu, elders are aware that the attitude and food habits of youth will determine their aging health, so educating young people remains the key. On both sides, elders wish to be healthy and active. They link old age with diseases as this period of life is one of illness and degeneration of multiple body functions. Thus, it is rather seen as a stage of life where they need to rest, be quiet and have a peaceful mind, which are deemed essential for the healthy aging. Elders require better prevention, control and regular monitoring of their health status in the health services (Wellman, 2007), but in Taabo the structural lack of functional social services and high healthcare costs result in limited health services utilisation, particularly affecting their psychological health (Gyasi, 2018). In Ebetsu, healthy ageing is partially link to these services and support.

### Perception of Elders as a Burden to Society

The study shows that the elders in both study sites have a dual perception of their role in the society. They see themselves as both a burden to their families and the next generation and as advisors and sources of wisdom for the stability of their families and communities. In Taabo, the reduction of their activities and their lack of physical activity lead to a considerable decrease in their income (which is, for the most part, derived from agricultural production). In this context, they become dependent on their children and family for the majority of their needs as an insurance system is lacking. In addition, the increasingly sedentary lifestyle exposes them to several diseases for which the support of their caregivers, especially of their family and children, are indispensable. The sedentary lifestyle of elders increases with their age and may affect their health status and leave them as burdens in terms of care (Kim and Lee, 2019). Protein is so deficient thus its access is reserved for the most affluent. Their physical, economic, and social suffering leads to the emotional psychological sufferings of the members of their social network (Berthe et al. 2013). In terms of their perception of their usefulness, elders emphasize that they are only a burden to their society. In contrast, they remain important advisors to their families and communities. They guide cultural practices in their community and are particularly indispensable in the social and political organization of their locality. According to Amyot (2016), in African traditions, an important role is given to elders through some tasks, which are especially devoted to them. An old man is an inspirer, a judge, a religious leader, a sacrificial priest, a decipherer of omens and dreams, and a poet, as do old women with invaluable experience in childcare, healer, marriage counselor, etc.

### Economic and Cultural Behaviors of Elders as Important Factor Affecting Healthy Aging

In the context of Taabo, there are no services for elders to prevent diseases among elders. They are often left to manage their own health, and they lack formal organization and education, thereby increasing their vulnerability. In Africa, the elders suffer from a lack of human, material, and financial resources and support (Berthe et al., 2013). African health systems could learn from the Japanese form of elders’ social organization. Japan is one of the models for an aging society. By looking at Japan’s model for elder management, it appears that the economic and cultural behaviors of elders are the most important factors affecting healthy aging. The current study corroborates the Japanese and the Swedish models, where one lives longer and healthier thanks to lifestyle, social status, diet, and heredity factors (Kim-Esnault, 2008). Encouraging the elderly to adopt a health-oriented diet and boost their collective organisation, as seen in Japan, would be an essential asset in promoting healthy lifestyle through sharing experience and awareness of the behaviour and physical requirements of ageing (Wellman et al., 2007). On the other hand, the family structure of older Japanese is dramatically changing because of increasing levels of non-marriage, childlessness, and divorce. Although at a limited level, lower emotional well-being is observed in elders who do not live with children (Raymo et al., 2008). Dependency of elders on their children in Taabo may be associated with better emotional well-being, and this value should be separately considered to discuss health in general.

### Awareness, Diet Dynamics and Health Outcomes

Most elders in Ebetsu are aware of what constitutes a healthy diet. They have the capacity to measure quantities and recall the foods they had. They have exact knowledge on the food composition they must select, based on their need to avoid overnutrition. They have concerns about the food freshness, the cost of aging to the society, and raise the issue of their autonomy. Their knowledge was expressed both in the choice of the products and the trust they place in local food, which is of high quality.

In the context of Taboo, the regular consumption of certain types of meat such as red meat and poultry is perceived as a sign of wealth and social success. However, the consumption of these proteins and certain beverages (alcoholic beverages and wine) remains sporadic among most elderly people and is mainly observed during celebrations and social events (weddings, baptisms, etc.). Obesity and arthritis have been identified as a common burden among elders, leading to several disabilities and NCDs, which reinforces their dependence and the burden of care for their families (Boateng et al., 2017). Regarding the dietary dynamics of the elders in Taabo, we notice that there is a strong reduction in the rate of eating with age in the context of Taabo. Most of the elderly people strongly emphasized the decrease in their appetite and the quantity of what they eat now compared to when they were young. According to the elders, this is explained by the emergence of many health problems that they face and by the decrease in the vitality of their organs which are increasingly affected by age. That is not the case in the Japanese context due to the traditional diet improved by umami taste (Mese and Matsuo 2007). This property enhances food palatability and protects the proper functioning of taste sensation. Thus, umami taste stimulation has been employed therapeutically to improve the flow of salivary secretion in elderly patients who have deficient umami taste sensation to avoid poor appetite, weight loss, and poor general health (Sasano et al., 2014). Nevertheless, in Taabo, despite this decrease in eating patterns, elders’ feeding habits from youth remain constant until old age. This reality leads in contexts of poverty and reduction in agricultural production to malnutrition and many other eating disorders. About malnutrition, elders defined malnutrition as undernourishment described by inadequate food intake, poor appetite, and loss of muscle and weight. We found two differences between the two groups of participants. The differences are in terms of food quality and food quantity. It has been shown that an incorrect diet facilitates the development of many non-contagious and chronic diseases, such as obesity, atherosclerosis, and type II diabetes, thus considerably impairing the quality of life (Ferry et al., 2005; Malazonia et al., 2021). Another consequence of malnutrition is associated with diseases, social and financial conditions, and frequent hospitalization (Arellano et al., 2004). In view of the eating habits and frequency of consumption of elders in Japan, we observe that they have a diversified diet with moderate quantities (Yokoyama et al., 2019). In view of the eating habits and frequency of consumption of elders in Japan, we observe that they have a diversified diet with moderate quantities. The presence of protein foods such as mutton and especially soya allows them to avoid protein malnutrition but above all to run less risk of metabolic and cardiovascular diseases. Because red meat and particularly processed meats are high in cholesterol and saturated and solid fatty acids, they tend to reduce it at an older age as it is perceived as one of the major risk factors for metabolic disorders. At the same time umami is involved in the regulation of various gastrointestinal functions (Prescott-Allen, 2001). This could partially explain why there is no need in Japanese traditional diets to use large amounts of animal fat or meats for optimal palatability—the meat-like sensation of traditional Japanese dishes with umami is sufficient. Also, small bites, due to the use of chopsticks, together with the combination of foods inside the mouth seem to contribute to satiety. But in Taabo, eating with the hand may allow huge quantities at the same time, compromising digestion. There is evidence showing that multiple alternations of foods decrease food consumption at the end of the meal (Brondel et al., 2009). The relatively small portion size of the main and side dishes is another trait that helps to avoid overeating since studies have shown that big portions encourage the consumption of larger meals (Ello-Martin et al., 2005). Several studies demonstrated a possible association between red meat consumption and cardiovascular disease (Medeiros et al., 2019). On red meat replace by product from soy, this seem to be in the same way of the results from corrected chemical index of the digestibility of different food proteins of FAO/WHO expert in 1991 who claimed that isolated soy protein is equal to casein, albumen protein. This diet is associated with a very low risk of cardiovascular disease as known to reduce blood pressure and blood glucose (Rivas et al., 2002). All features of the scheme seem to participate in this prevention is confirmed by Okamoto et al. (1995) and Mimura et al. (1992). Elders paid much attention on their health by consuming less salt, sugar, alcohol, fatty food, and red meat (beef and lamb) that reduce the risks of developing non-communicable diseases, cardiovascular diseases, osteoporosis, cancer, high blood pressure, and diabetes. Their traditional dietary cultures help them in that way, leading UNESCO to list them as part of Intangible Cultural Heritage. In contrast a different picture is observed in Taabo where high-energy diet intake not only exposes them to overweight and obesity but also to the risk of protein malnutrition due to the protein deficiency in their meals (Mattes 2014). They consumed what they find, not really what they want because they depend on the family diet. Despite their specific nutritional needs that is determined by age-related physiological and pathological changes (Mattes 2014). A model of nutrition in the elderly people is very particular, considering dietary requirements, eating patterns, and dependence for eating skills (Aune et al., 2017). Elders in Taabo could not fulfill this requirement because no pension or specific assistance to them exist. In practice, many social assistance programs in developing countries show system that operate and exclude elders and their households (Holzmann et al., 2009). Elders in Taabo, contrary to the Japanese, did not pay attention to the quality of their diet because of lack of nutrition education at a younger age and most of them did link nutritional patterns with health.

The limitation of the current comparative qualitative study is the multiple translations that could introduce biases and misinterpretation. While we observe precise knowledge on diet from women, the gender aspect was not analyzed. Elders raised their relationship with youth and social services, but this triangulation was not performed to better discuss the results.

The main learnings from the current study are the importance of diversified and quality food that match the physiological need, the physical activity that allows active aging, the need for family network and intergeneration dialog for enhancing good mental health, adapted social services to elders, nutritional education for youth.

### Limitations of the Study

In this study, the major limitation was the insufficient quantitative data from Taabo and to generate strong statistics from both sites’ focus group discussions, o our main aim being to understand the healthy aging perception. But the qualitative approach has advantages and power to explain the meaning of diet parameters and health concepts. The process of translation between languages (Japanese to English and French to English) can be a source of bias in capturing the meaning of words, phrases, proverbs, and sentences. Another limitation of the study is related to the participants’ selection. Elders who were unable to express themselves due to illness or unable to move were not included in the study. Also, the age criteria used in Ebetsu were not met in Taabo. The age range was 60–90 years in Ebetsu and 55–80 years in Taabo. This difference is explained by the difference in the low life expectancy and proportion of elderly people in the Taabo population. The life expectancy of the population in Japan is higher than in Côte d'Ivoire. Finally, we can point out a difference between the care system and support conditions of the elders in Taabo compared to Ebetsu. In Taabo, the elders mostly live in families around their relatives. Therefore, it is sometimes difficult to bring together several elders in a systematic way for group activities such as focus groups or to assess the quality and quantity of their diet when they eat in groups.

## Conclusion

This comparative study between the elderly in Japan and Côte d'Ivoire highlighted challenges that need to be addressed to ensure healthy aging of the populations in both contexts. This requires a synergy of action from different sectors such as health systems, agricultural systems and nutritional education. From a young age, it is important to adopt healthy dietary habits such as breakfast, sufficient vegetable intake and less fat, sugar and salt intake, combined with balanced protein-energy consumption. Aging is culturally rooted and embedded in the language expression that describes the consumption pattern. The empowerment of elders through genuine social assistance that allows them to make the best decision for their diet and health is important. Healthy agriculture that produces diversified food may offer a large range of fresh food. The perceptions and the social meanings associated with aging and feeding, including the habits, dietary practices, and social prohibitions that result, also need to be considered to promote adherence to new attitudes related to a healthy diet and physical activity for healthy aging. Elders are vulnerable and need support with adapted nutritional and social services. Research should more prominently address the elder nutrition questions and communication tools for healthy aging (Gordon et al., 1977; FAO, 1991; Martin, 2001; Ashima, 2004; Breuil and Euller-Ziegler, 2004; Ferry et al., 2005; Wellman, 2007; Wellman et al., 2007; Ferry, 2010; Aune et al., 2017; Hache and Bourcet, 2017; Ménard, 2021).

## Data Availability

The original contributions presented in the study are included in the article/Supplementary Material, further inquiries can be directed to the corresponding author.
