# Supplementary material for: Healthy Aging: Comparative Analysis of Local Perception and Diet in Two Health Districts of Côte d’Ivoire and Japan
Source: Front Aging. 2022 Apr 25;3:817371. doi: 10.3389/fragi.2022.817371 (PMC9261373; doi:10.3389/fragi.2022.817371)
Supplement: Supplementary file 1 [file Table1.DOCX]

# **Quantitative information N° ___**

Date : __________________________________________

Residence __________________________________________

Origin __________________________________________

## **General information**

Profession: __________________________________________

Age ____________

Sex

| M |  |
| --- | --- |
| F |  |

Smoker

| Former |  |
| --- | --- |
| Current |  |
| No |  |

## **Wealth status**

| High |  |
| --- | --- |
| Medium |  |
| Low |  |

## **Body condition**

| Weight (Kg) |  | |
| --- | --- | --- |
| Overweight |  |  |
| Obese |  |  |
| Medium |  |  |
| Low |  |  |

Time for physical activities: ______________________________________

Past dietary practice ______________________________________

## **Illness**

Known important disease: ______________________________________

| Presence of illness |  |
| --- | --- |
| Absence of illness |  |
| Infections (chronic lung diseases) |  |
| Hypertension |  |
| Cardiovascular diseases |  |
| Diabetes |  |
| Cancer |  |
|  |  |

## **Diet**

### 5.1 Frequency

| Processed food |  |
| --- | --- |
| Prepared food |  |

### **5.2 24h recall**

| Processed food |  |
| --- | --- |
| Prepared food |  |

### **5.3 Food intake (frequency)**

| **Nutrients (nutrition variables)** | **Time per period (day, week, month)** |
| --- | --- |
| Processed food |  |
| Cereals |  |
| Potatoes and starches |  |
| Sugars and sweeteners |  |
| Confectioneries |  |
| Fats and oils |  |
| Nuts and seeds |  |
| Pulses |  |
| Fish and selfish |  |
| Meats |  |
| Eggs |  |
| Milks, dairy products |  |
| Vegetables |  |
| Fruits |  |
| Fungi, mushrooms |  |
| Algae |  |
| Beverages tea |  |
| Beverages Soda |  |
| Beverages Alcohol |  |
| Seasonings &spices |  |

### **Food intake (quantity)**

| **Nutrients (nutrition variables)** | **Weights prepared or purchased (g)** | **Quantity/proportion consumed (g, all, ½, 1/3, 1/4 )** | **Observation** |
| --- | --- | --- | --- |
| Cereals |  |  |  |
| Potatoes and starches |  |  |  |
| Sugars and sweeteners |  |  |  |
| Confectioneries |  |  |  |
| Fats and oils |  |  |  |
| Nuts and seeds |  |  |  |
| Pulses |  |  |  |
| Fish and selfish |  |  |  |
| Meats |  |  |  |
| Eggs |  |  |  |
| Milks, dairy products |  |  |  |
| Vegetables |  |  |  |
| Fruits |  |  |  |
| Fungi, mushrooms |  |  |  |
| Algae |  |  |  |
| Beverages tea |  |  |  |
| Beverages Soda |  |  |  |
| Beverages Alcohol |  |  |  |
| Seasonings &spices |  |  |  |
| Processed food |  |  |  |

General observations:

______________________________________________________________________________________________________________________________________________________________________________________________________________________________________________________
