# Supplementary material for: Healthy Aging: Comparative Analysis of Local Perception and Diet in Two Health Districts of Côte d’Ivoire and Japan
Source: Front Aging. 2022 Apr 25;3:817371. doi: 10.3389/fragi.2022.817371 (PMC9261373; doi:10.3389/fragi.2022.817371)
Supplement: Supplementary file 2 [file Table2.DOCX]

**Appendix: Guide for Focus Group Discussion (ex. Ebetsu)**

**1. Selection of participants, organisation**

- Site: Ebetsu, Sapporo, Hokkaido
- Population: 9-10 peoples on 2 sites
- Age > 60 years (age of retirement?)
- Language: Japanese; men (4), women (5); venue: to be defined; duration: 1 hour

**2. Introduction**

- Presentation of participants, presentation of the project, consent form, timing, recording authorization
  - Japan is an example of country where people **live longer**
  - Confirmation (why they think Japan is an example of country where people live longer?)/ What are the main **reason**?
  - **Perception** on ageing? Which factors affect the variation of age process (gender, origin, profession, …)
- Organisation, services
  - Positions of elder **in the society**, what should it be?
  - How **families organised** around ageing?
  - **Services** provided/ supporting elders?
  - Main **challenges** or problems of elders?/ Socio-economic cost of ageing?

**3. Perception on healthy ageing**

- Condition for healthy ageing?
- Activities of elders?
- Health problems (main pathologies)?
- Health system around ageing?
- Special care of elders?
- How they access services?
- What are their own hygiene of life?
- Ageing risks and outcomes

**4. Diet of elders**

- What is the healthy diet? What elder eat, why? What do they avoid, why?
- What are the restrictions, why? (ageing, health, culture)
- Specific type of elder’s diet/ Composition and perceived role of each component
- Calories estimates/ Supplements
- Access and effectiveness of diets/ Cost estimate or evaluation

**5. Animal source food and NCDs**

- Animal source food
  - Red meat (cow, sheep, goat, sausages,..)/ White meat (poultry, pork…)/ Dairy products (butter, milk, cheese…)/ Fish/ Sea food/ Subtitutes (e.g. soya,…)
  - Consumption patterns (consensus on portion and quantities)/ Quantity/ Quality/ Frequency
- Non communicable diseases (Diabetes, HTA, …)
- Motivation, incentives for elders ?

**6. General questions**

- - What is the animal source food consumption pattern of elders?
  - What are the main determinants of change in diet of elders?
  - Under which circumstances there is a change in the animal source food diet of elders?
  - Which incentives contribute to nutritional behaviour change and decision making among elders?
  - What is the link between the emergence of non-communicable disease and the change in animal source food intake?
